# 838. The Role of Surgery in the Management of Invasive Fungal Rhinosinusitis Complicated by Orbitocranial Extension in the Era of Azole Antifungals

**DOI:** 10.1093/ofid/ofad500.883

**Published:** 2023-11-27

**Authors:** Idit Tessler, Rachel Shemesh, Gilad Sherman, Ethan Soudry, Sharon C Chen, Oren Ziv, Sofia Kordeluk, Dvir Yohai Bar-On, Ilya Novikov, Arkadi Yakirevitch

**Affiliations:** Sheba Medical Center, Ramat Gan, Tel Aviv, Israel; sheba medical center, Ramat-Gan, Tel Aviv, Israel; The Edmond and Lily Safra Children's Hospital, Sheba Medical Center, Kiryat Ono, HaMerkaz, Israel; Rabin Medical Center, Tel Aviv, Tel Aviv, Israel; Westmead Hospital, Sydney, New South Wales, Australia; Soroka medical center, Beer sheva, HaDarom, Israel; Soroka Medical Center, BEER SHEVA, HaDarom, Israel; Rabin Medical Center, Tel Aviv, Tel Aviv, Israel; The Gertner Institute for Health Policy and Epidemiology, Sheba Medical Center, Beer-Sheva, HaDarom, Israel; Sheba medical Center, Kiryat Ono, HaMerkaz, Israel

## Abstract

**Background:**

Orbitocranial fungal infection (OCFI) is a potentially lethal complication of fungal rhinosinusitis, historically requiring aggressive surgery despite its significant morbidity. Over the past two decades, early use of azole agents has been introduced to support amphotericin B formulations as first-line treatment followed by step down therapy with azoles, demonstrating promising outcomes. To date, there is no data regarding the contribution of surgery in the “azole era” to patients’ survival. We aimed to provide real-life data on azole treatment outcomes and the role of surgery in the current management of OCFI.

**Methods:**

Data was collected retrospectively from a chart review of the four tertiary participating centers in Israel and Australia between January 1, 2009 and January 1 2020, as well as additional cases identified through a systematic literature review (Fig. 1). The study group included patients with OCFI treated with azole antifungals. Control cases were all treated with amphotericin B. The cranial and orbital involvement was staged based on imaging (Fig. 2). The extent of surgical resection was also classified to allow for inter-group comparison.

Flowchart for identification, screening, eligibility, and inclusion for the systematic review according to the Preferred Reporting Items for Systematic Reviews and Meta-Analyses (PRISMA)

**Figure:**
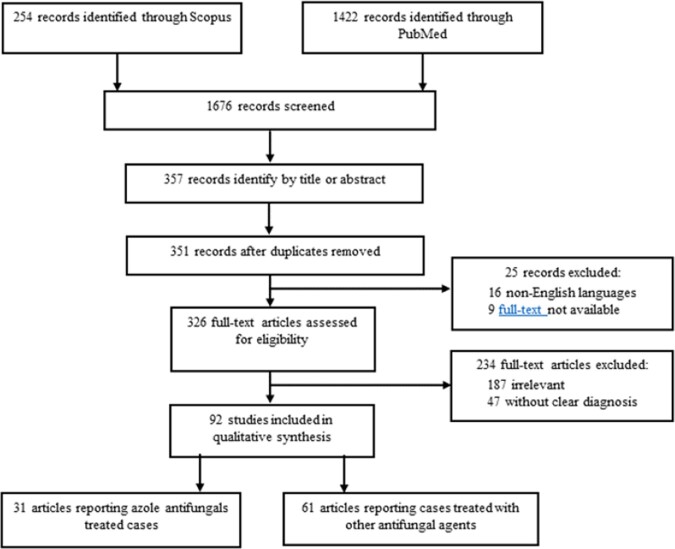

Staging of orbitocranial fungal infection used in the study and T1 contrast-enhanced magnetic resonance images illustrating each stage

**Figure:**
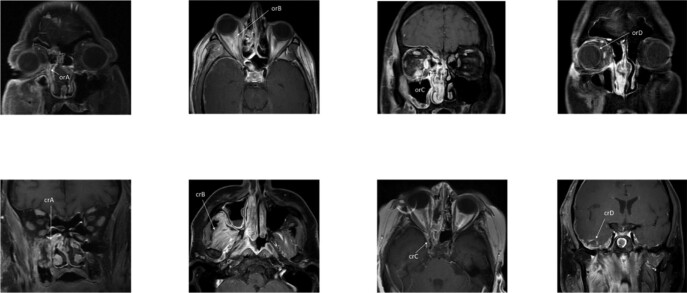

I. Orbital extent: orA - periorbit, lacrimal apparatus; orB - orbital fat; orC - oculomotor muscles; orD – optic nerve, eyeball. II. Cranial extent: crA - pterygopalatine fossa, nasopharynx; crB – temporal/infratemporal fossa; crC – cavernous sinus, dura; crD – brain, spinal cord.

**Results:**

There were 125 patients in the azole anti-fungal (AAF) group and 153 in the control group (Table 1). Mucorales and *Aspergillus* were identified in 28% and 72% of AAF cases, respectively, and 67% and 33% of control cases, respectively (p< 0.001). Among patients with cranial extension, 22.6% were operated on in the AAF group and 18.4% in the control group (Table 2). However, meninges and brain resection were performed only in the controls (10.5%) and never in the azole antifungals group (p=0.045). Orbital involvement required surgery in 25.9% of AAF cases and 38.6% of controls (p=0.12). Despite a more aggressive cranial involvement, azole-treated patients’ 3-year mortality was significantly lower than controls (21% vs. 52%, HR 0.27, p< 0.001, Fig. 4).

Comparison of the study groups according to the orbitocranial fungal infection extent

**Figure:**
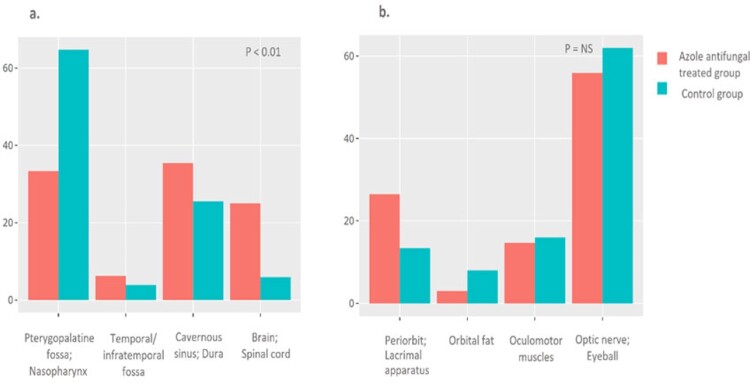

a. cranial extent; b. orbital extent

Kaplan Meier curve on the orbitocranial fungal infection specific survival during up to 3 years of follow-up

**Figure:**
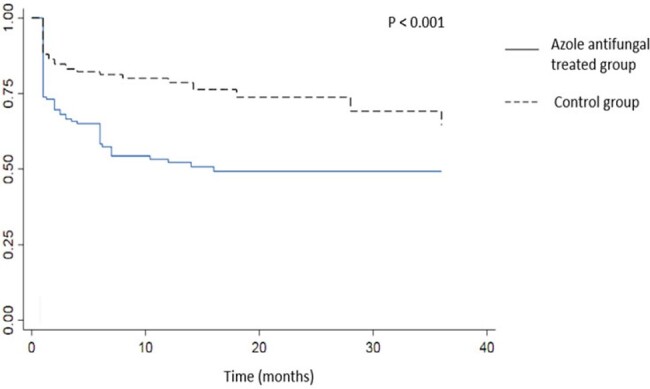

*Categorical variables are presented in n (%); **Continuous variables are represented as mean (±SD); ªAcquired immunodeficiency syndrome; bHematologic disease leading to immune compromised status; *Including 6 patients with both Mucorales and Aspergillus

Univariate analysis of the study groups' main characteristics

**Figure:**
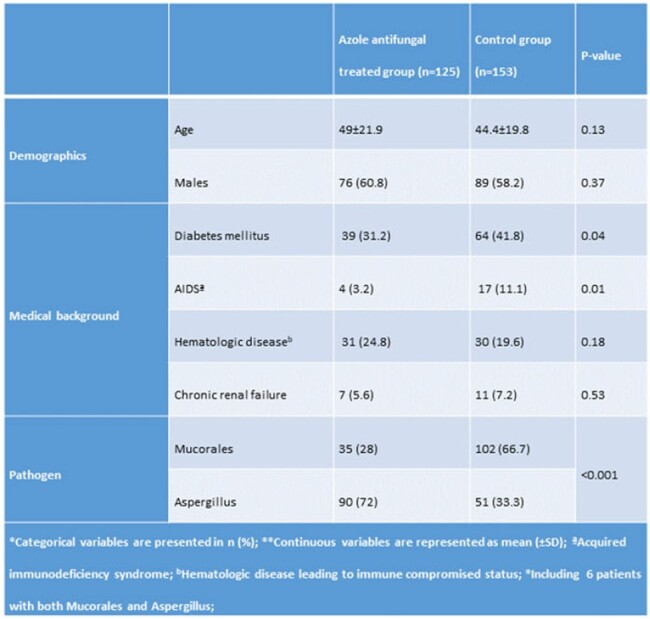

**Conclusion:**

Despite less aggressive surgical intervention for cranial involvement, OCFI patients treated with azoles had a higher survival rate, suggesting we may improve morbidity with a more conservative surgical approach in conjunction with azole treatment. The same trend is emerging for orbital involvement.

Surgery for rhino-orbital-cerebral mycosis patients.

**Figure:**
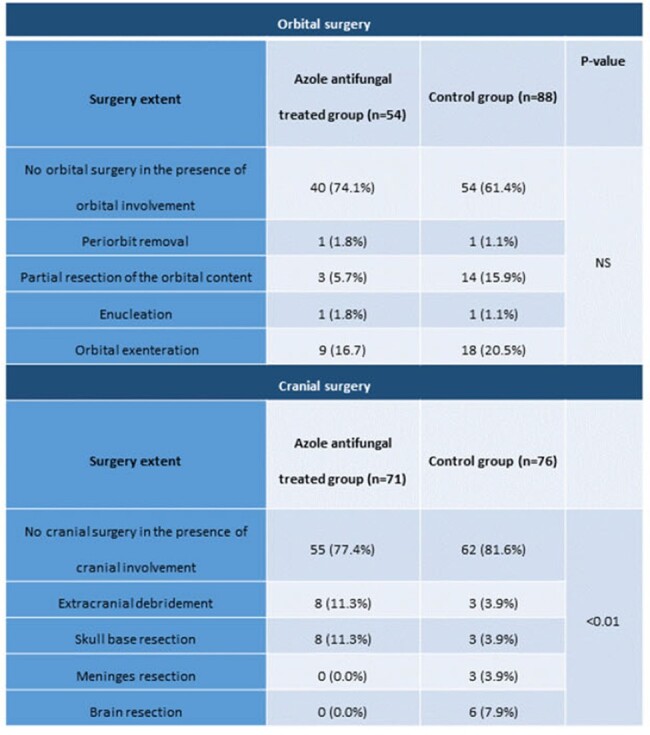

**Disclosures:**

**Sharon C. Chen, PhD MBBS**, F2G PTy LTd: Advisor/Consultant|F2G PTy LTd: Grant/Research Support

